# Two hundred years of historical spawning and nursery data for coregonine fishes in the Laurentian Great Lakes

**DOI:** 10.1038/s41597-026-06974-1

**Published:** 2026-05-07

**Authors:** Cory O. Brant, Sofia Silvis, David H. Bennion, Chris Castiglione, Kieran Tyrrell, Karissa Hannahs, Michael Slattery, David Bunnell, Andrew Honsey, Ralph Tingley, Katelyn King, Karen M. Alofs, Amanda Ackiss, Charles R. Bronte, Jason Smith, Matthew Herbert

**Affiliations:** 1 U.S. Geological Survey, Great Lakes Science Center, 1451 Green Rd., Ann Arbor, MI 48105 USA; 2 U.S. Fish and Wildlife Service Lower Great Lakes Fish and Wildlife Conservation Office, 1101 Casey Road, Basom, NY 14013 USA; 3 U.S. Geological Survey, Great Lakes Science Center, Tunison Laboratory of Aquatic Science, 3075 Gracie Road, Cortland, NY 13045 USA; 4 U.S. Geological Survey, Great Lakes Science Center, Hammond Bay Biological Station, 11188 Ray Road, Millersburg, MI 49759 USA; 5 University of Michigan, School for Environment and Sustainability, 440 Church St., Ann Arbor, MI 48109 USA; 6 U.S. Fish and Wildlife Service Green Bay Fish and Wildlife Conservation Office, 2661 Scott Tower Drive, New Franken, WI 54229 USA; 7 Bay Mills Indian Community, 12140 W. Lakeshore Drive, Brimley, MI 49715 USA; 8 The Nature Conservancy, 101 E César E. Chávez Ave, Lansing, MI 48906 USA

**Keywords:** Biodiversity, Freshwater ecology

## Abstract

Historical data can provide critical ecological information for species across the globe, many of which are facing unprecedented rates of ecosystem change. Yet, historical information related to freshwater species, especially fishes, remains scattered, often in original formats, and underutilized for informing conservation and restoration activities. Here, we present a Data Descriptor called Coregonine Spawning History (CORHIST), a database designed to house diverse data related to past spawning and nursery areas for fishes in the family Salmonidae, subfamily Coregoninae (ciscoes and whitefishes), in the Laurentian Great Lakes and their tributaries. Data for 11 species of coregonines historically occurring in the Great Lakes are included in CORHIST. Over 3,400 occurrence records at the coordinate scale have been entered, over 2,200 of which are for Cisco (*Coregonus artedi*) and Lake Whitefish (*C. clupeaformis*)—two focal species for which there is either multinational conservation interest or restoration efforts underway in the Laurentian Great Lakes. CORHIST is already proving useful for several studies developing habitat suitability models and delineating spatial units for conservation or restoration planning.

## Background & Summary

Award-winning writer and humanitarian Pearl S. Buck once said, “If you want to understand today, you have to search yesterday,” and this sentiment is especially true in the world of ecology. History plays a vital role in conservation science and species management^1–3^. When properly organized, scientists, managers, and communities can use historical data to better understand past abundances, where critical habitats once occurred, past requirements for species of concern, and other important context for present day observations^4–8^. For historical data to be useful for conservation problems, diverse data and ways of knowing, from first-hand accounts and Indigenous Ecological Knowledge (IEK) to scientific surveys and much more, must be preserved, indexed, and shared among communities. Among many advantages, accessible historical data can protect new generations of scientists and stakeholders against Shifting Baselines Syndrome, a phenomenon highlighted by Pauly^9^ but long known by Indigenous Communities^10,11^, when describing the intergenerational loss of knowledge regarding past environmental and ecological conditions for species that are exploited, extirpated, or extinct. Historical information that is accessible and inclusive of diverse data types and ways of knowing can help regain that lost knowledge by keeping baseline information accessible for new generations of scientists, managers, and communities^12,13^.

Historical data becomes increasingly important in systems where long-term data sets are sparse and records are variable in resolution and accessibility^5,12,14^, and this is especially true for ciscoes and whitefishes (family Salmonidae, subfamily Coregoninae) in the Laurentian Great Lakes^15^. Historically, 11 named species of coregonines were recorded in the Great Lakes, including three species of whitefishes (Lake Whitefish *Coregonus clupeaformis*, Round Whitefish *Prosopium cylindraceum*, and Pygmy Whitefish *P. coulterii*) and eight species of ciscoes (Cisco or Lake Herring *C. artedi*, Shortjaw Cisco *C. zenithicus*, Shortnose Cisco *C. reighardi*, Blackfin Cisco *C. nigripinnis*, Bloater *C. hoyi*, Kiyi *C. kiyi*, Longjaw Cisco *C. alpenae*, and Deepwater Cisco *C. johannae*)^16,17^. Indigenous Peoples around the Great Lakes Basin (the Basin, herein) have relied upon coregonines sustainably for millennia for both sustenance and trade, sharing important cultural connections with Lake Whitefish (Adikameg, in Ojibwe Anishinaabemowin) and Cisco (Otoonapii, Odoonibiins, and many more names)^18,19^. Both Cisco and Lake Whitefish exhibited large spawning runs each fall into nearshore areas^15,20^, tributaries^21,22^, and offshore areas^23^. Within just a century through the Industrial Revolution and lumber eras, multiple threats including pollution and damming^22,24^, invasive species impacts^25–28^, and intensive selective exploitation^8,29,30^ rapidly drove extinction of at least two deepwater Cisco species (*C. johannae* and *C. alpenae*) and lake-specific extirpation of several more^31^. Prior to 1960, little to no regulation was directed toward protection of coregonines from aggregation and interception fisheries in the fall and early winter^24^, in many cases resulting in peak extractive rates at well-known spawning grounds just before or during spawning seasons^32^. In part due to a popular market for Great Lakes coregonines^29^, a wide array of records began to emerge for these species—including natural history survey data, commercial catch reports, oral histories, museum collections, annual surveys, newspaper stories that highlighted particularly large spawning runs, and much more.

While historical information related to coregonine occurrence, spawning locations, and other habitat preferences exists, much of it is heterogenous in accuracy and resolution, and much remains inaccessible for mapping or other analyses (*i.e*., not digitized or translated into a machine-readable format), making most of these records difficult to utilize for conservation or restoration actions. Beginning in 2020, our data team consisting of spatial and data scientists, ecologists, biologists, and historians, assembled to create the Coregonine Spawning History (CORHIST) database^1^. Our team focused on spawning and nursery occurrence data for CORHIST because spawning and nursery grounds are feasible target areas for conservation, restoration, and monitoring, are mappable, and are often described in detail in historical sources and through IEK^19,20,33^. Isolated by the global pandemic, our team held virtual calls weekly for several years to discuss historical sources and database structure, and to test database fields by indexing historical records. Inclusion of diverse data types has been shown to improve both temporal and spatial resolution of studies aimed at identifying essential fish habitats^34^. We therefore did not restrict ourselves to searching for fisheries-dependent data alone, or independent data alone, for example. Nor did we restrict ourselves to searching for any specific format of source or knowledge. Over 500 original or primary sources (cited in SOURCE reference table, online), including field notes, journals, commercial and recreational catch reports, ship logs, raw data sheets, peer-reviewed manuscripts, interview transcripts, photographs, and more, were collected and reviewed for evidence of spawning and/or nursery occurrence records. Sources were discovered in a variety of locations including open-access online archives (*e.g*., National Archives, HathiTrust, Biodiversity Heritage Library, local libraries, university collections) as well as various field stations and museums visited locally and across the Basin.

**Fig. 1:**
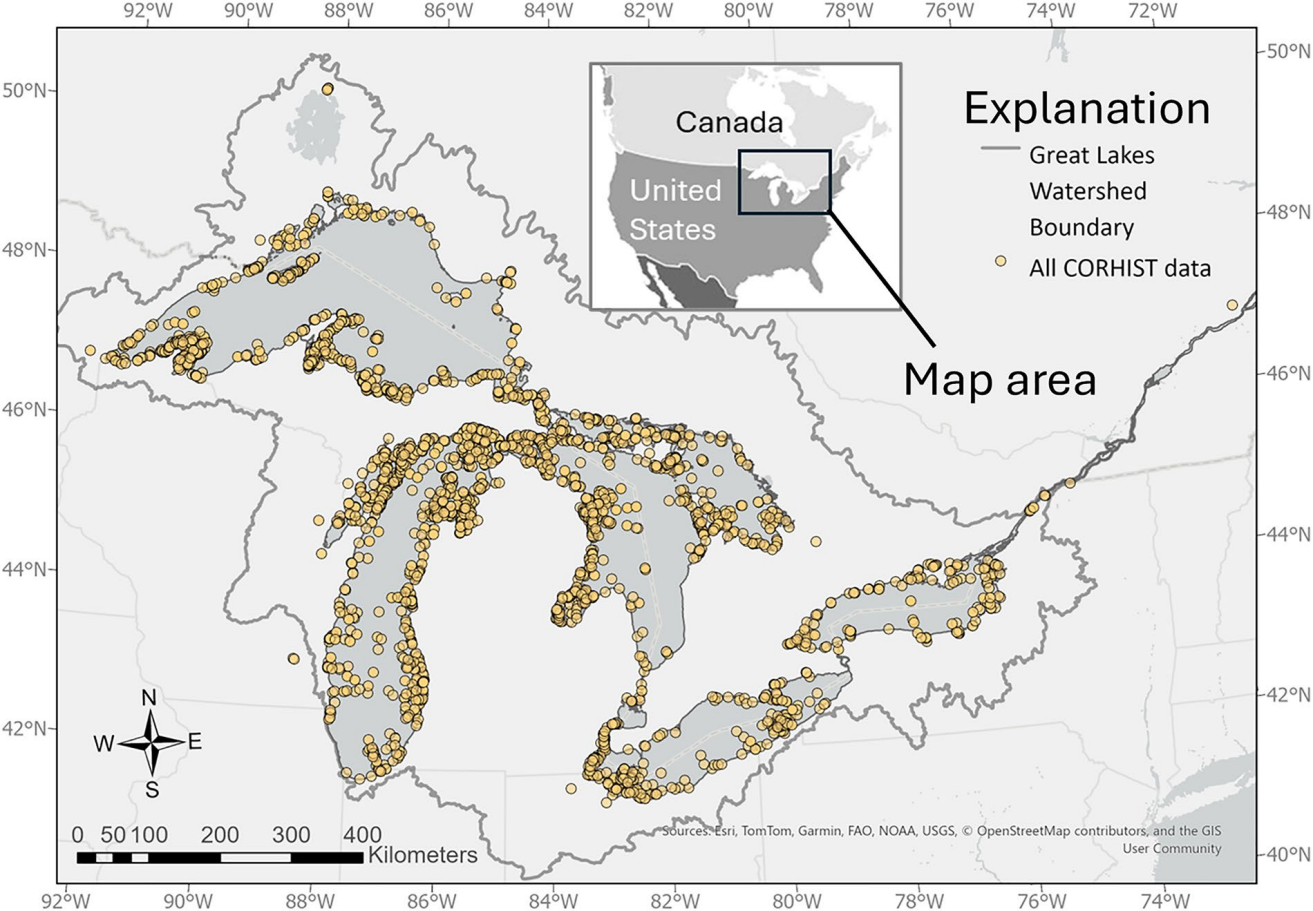
A map of the Great Lakes and surrounding watershed boundary showing locations of all CORHIST records (orange points). The dark gray boundary denotes the Great Lakes watershed (watershed boundary source: GLAHF; https://www.glahf.org/watersheds/) and light dashed line the U.S. – Canada boundary.

**Fig. 2:**
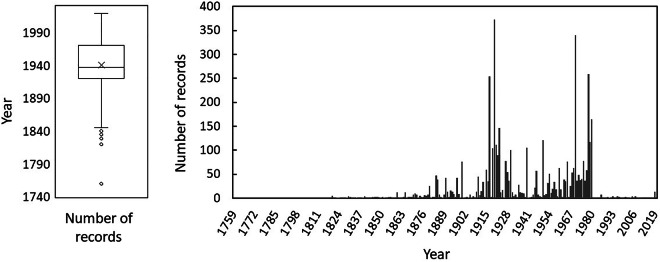
Relationships between the year of each observation and the number of records in CORHIST. Records are first displayed as an overall mean of years, then as the number of records across years.

**Fig. 3:**
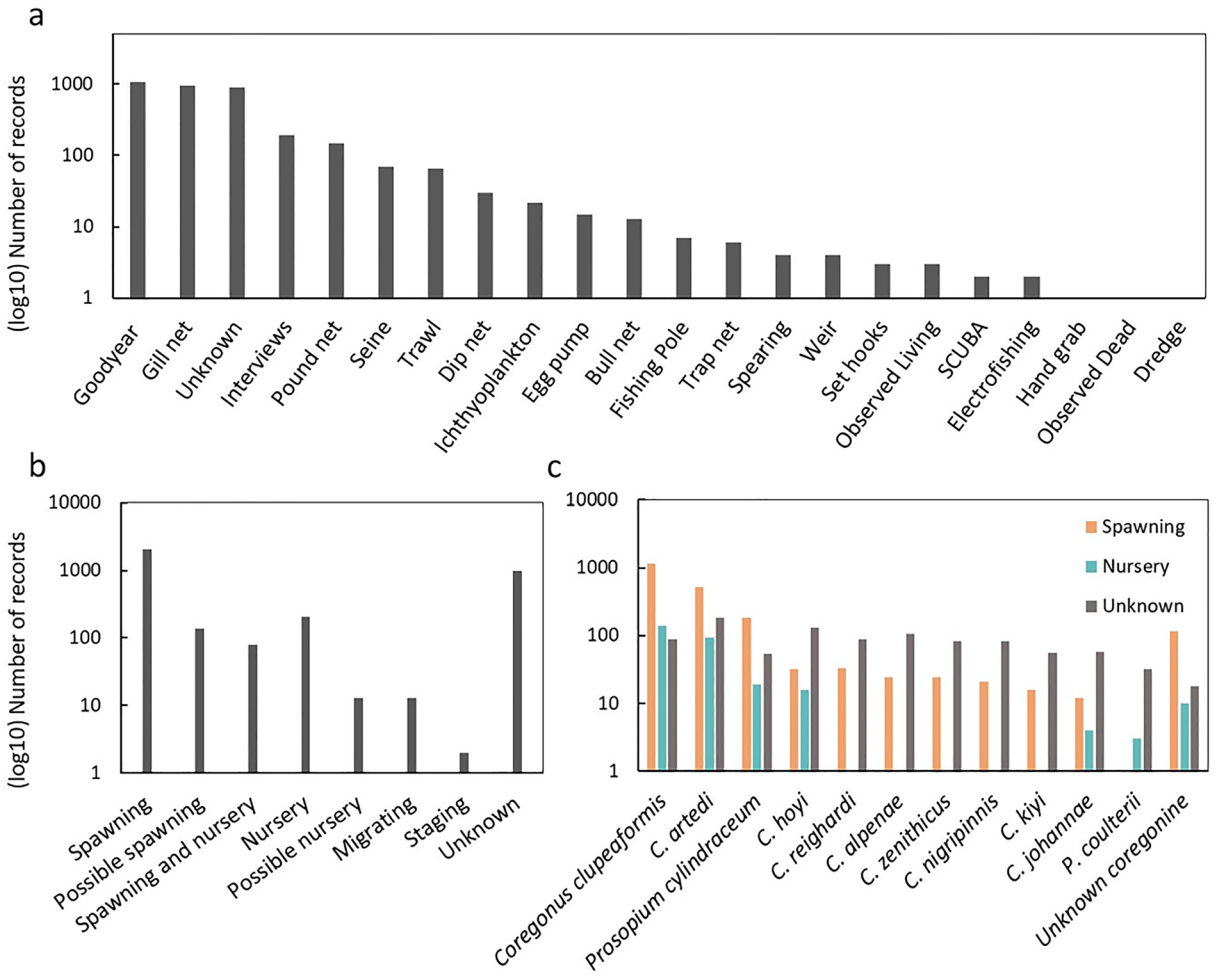
Relationships between the number of records (log scale) in CORHIST and several popular fields in the database. Graph (**a**) shows the number of records by SAMPLE_TYPE, (**b**) shows the number of records by SPAWNING_CLASSIFICATION, and (**c**) shows the number of records for each SPECIES separated by Spawning (orange), Nursery (aquamarine), and Unknown (gray) SPAWNING_CLASSIFICATION designations. All fields are defined in the metadata dictionary (online only).

During this study, 3,478 occurrence records for coregonines were discovered across all five Great Lakes, including their connecting channels and tributaries. While spatial coverage is overall relatively homogenous throughout the Basin (Fig. 1), many offshore areas in the largest lakes were, and still are today, rarely visited, hence observations are few. Additionally, the life histories, including spawning locations and behaviors, are not well understood for deepwater coregonine species, many of which are now locally extirpated or extinct^15,16^, further contributing to very few records discovered in offshore zones (Fig. 1). CORHIST database observations span from 1760 to 2007, with most observations falling into the 1920s – 70 s (Fig. 2). Spawning atlas maps such as those in Goodyear *et al*.^20^, as well as gill netting records, published interviews, and other commercial fishing gears were some of the most common sampling types (see SAMPLE_TYPE reference table^1^, online only) that yielded coregonine occurrence records (Fig. 3a).

Over 2,500 records included sufficient evidence for classification as *Spawning* and/or *Nursery* areas, both of which were designations determined by our expert working group. Caveats related to our spawning or developmental status designations for each record in CORHIST will be expanded upon in the Usage Notes section of this manuscript. Briefly, all records in the CORHIST dataset were classified related to a spawning or developmental status using the evidence found in original sources along with the defined terms in both the SPAWNING_CLASSIFICATION and SPAWNING_CONDITION reference tables^1^ (online only). Numeric codes from these reference tables were added to the dataset to indicate our assignment of a spawning status to each record. It should be understood by users that these designations were assumed, based on evidence, by our team but are subject to interpretation. Users can therefore choose to use our spawning status designations or refer to original sources, which are all publicly available and linked with each record, to make their own independent designations. To be designated as a *Spawning* occurrence (numeric code 1 in the SPAWNING_CLASSIFICATION reference table), our group agreed at the beginning of the study that the original source would need to state directly that the location described was a spawning area or, if physical fish were examined as part of the record, the record would need to show evidence that specimens were in spawning condition based on terms defined in the SPAWNING_CONDITION reference table (*e.g*., ripe, gravid, emitting milt/eggs, spawning, and several more). Terms in the SPAWNING_CONDITION reference table^1^ were extracted from the literature, mainly from Koelz^16^ and Brown-Peterson *et al*.^35^ while the terms in the SPAWNING_CLASSIFICATION reference table were created and defined by our working group. The numeric code for each term that was identified in the original source is included in respective fields in the CORHIST dataset, so users can see why we decided a record was a *Spawning* record. For a *Possible Spawning* designation (numeric code 2 in the SPAWNING_CLASSIFICATION reference table^1^, online only), our group had to discover some spawning evidence in the original source, such as notably large catches during spawning months, but no direct statement or physiological evidence that these fish were spawning could be found. For a *Nursery* occurrence designation, our group needed to locate evidence in the original source that larval or juvenile specimens were captured and described at a location, or the original source would need to directly define the area as a nursery area for the species. *Possible Nursery* designations then followed similar rules as *Possible Spawning* designations, where no direct evidence for a nursery was discovered in original sources, but indirect evidence that captured specimens were small (within juvenile size ranges). *A Spawning* designation is the most common designation in the dataset (Fig. 3b). *Possible Spawning* was the 2^nd^ most common. Well over half (2,195) of the occurrence records in CORHIST are for Lake Whitefish and Cisco, making them the most common species found in historical sources (Fig. 3c).

The CORHIST database has already proven integral for the planning and implementation phases of a Basin-wide Coregonine Restoration Framework, or CRF, an adaptive and multi-national effort aimed at conserving and restoring coregonines in the Great Lakes^31^. Within the CRF, the CORHIST database has informed species distribution models and maps aimed at describing and comparing historical versus contemporary spawning locations^36^. CORHIST and derived models and maps have been used for delineating spatial conservation units based on spawning grounds for key coregonines of interest^37^. Data in CORHIST informed a suite of recent proposals in the Great Lakes related to multinational Cooperative Science and Monitoring Initiative projects (CSMI, see https://greatlakescsmi.org/), including several contemporary coregonine population and early life history monitoring efforts on or near historical spawning grounds. The database structure of CORHIST will serve as a useful template for other research groups looking to collect and index historical biological data from diverse record types. At its foundation, the CORHIST database preserves and renders accessible knowledge related to spawning and nursery areas of coregonines for posterity, providing historical data for generations to come.

## Methods

### Sources and database fields

Inclusion of a wide variety of sources was deemed critical at the outset of this project since diverse data types have been shown to lead to more consistent estimations of important habitats^34^. Over five years we conducted a systematic and rigorous search in archives, museums, databases, and agency records to collect information on coregonine reproductive areas in all five Great Lakes. Spanning across the US-Canada border the Laurentian Great Lakes including Lake Superior, Lake Michigan, Lake Huron, Lake Erie, Lake Ontario, Lake Saint Clair, connecting channels, and several thousand tributaries, collectively cover more than 240,000 km^2^ to create one of the largest freshwater ecosystems on Earth. We did not include tributaries in our search, although tributary records were added to the CORHIST database if they were discovered. An equally rigorous search for Great Lakes tributary-specific coregonine spawning records remains a priority and knowledge gap in this dataset. Sources mainly included spawning atlases for a wide range of fish species (beyond coregonines) published in the 1980s or earlier^20,33,38,39^, ship logs and other field journals, typescript and notes related to museum specimens, government reports related to fisheries surveys, journal articles, newspaper clippings, published interview transcripts, and commercial catch reports collected by states and the Province of Ontario. Most of these sources were compiled in the context of various Great Lakes commercial fisheries, with an original purpose to provide a “status report” on species or populations of interest. Often these populations were already in decline by the time the research was conducted^24,29,32^. Subsequently, many of the records in CORHIST are near major cities or ports and contain data heavily skewed toward commercially popular species like Lake Trout (*Salvelinus namaycush*), Lake Whitefish, Cisco, and Yellow Perch (*Perca flavescens*). Given these biases, these sources do not represent true baseline ecological conditions across taxa, however they do provide valuable details related to the habitat use, location, population dynamics, and value of species of interest.

**Fig. 4:**
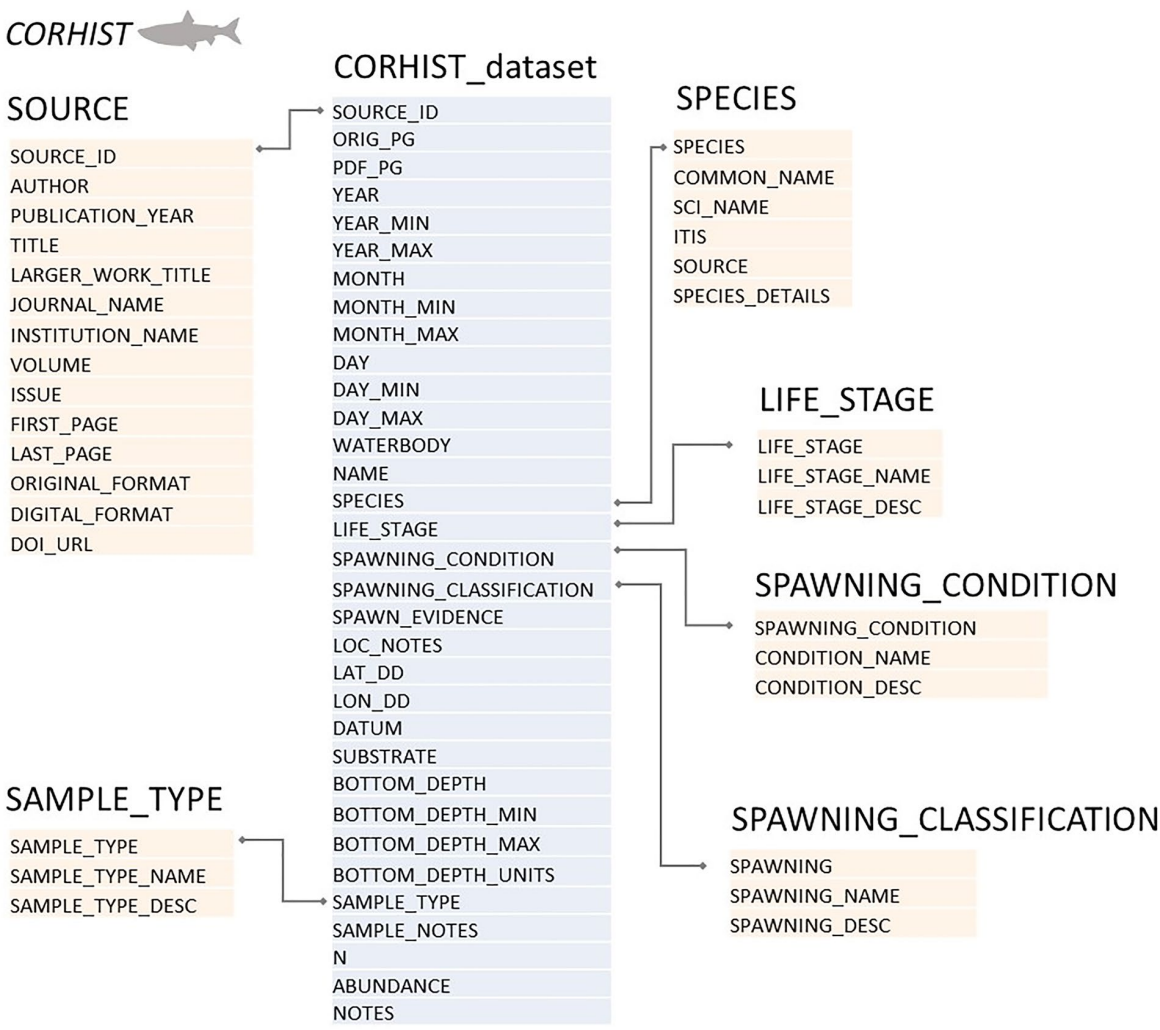
The entity relationship structure of CORHIST. The central box in light blue represents the main CORHIST_dataset. All other boxes (light peach) represent reference tables with headers indicating the name of each table and all field names listed below each header. All connections between primary and secondary keys are shown by gray lines. All field names are described in a metadata dictionary (online only).

Sources containing text were first scanned for keywords related to coregonine names, fishing activity, spawning, and other details related to a fishery. Our keywords included all species common and scientific names in the SPECIES reference table (online only), all names in the alternative names table (Supplemental Table 1), and all keywords listed in SPAWNING_CONDITION, SPAWNING_CLASSIFICATION, LIFE_STAGE, and SAMPLE_TYPE reference tables (online only). When information related to a potential spawning area, or nursery area, for a coregonine was located, the page numbers (digital PDF page and/or the original page number) were recorded, along with the date of the observation and any location notes, including waterbody and any additional descriptions of the fishing grounds or site name. Species notes, including the common and scientific name of the specimen observed were added, as well as notes related to evidence of spawning behaviors, life stages, or maturity including spawning condition. Finally, notes on gear types, water temperature, effort, substrate, quantitative and/or qualitative catch numbers, and any general notes were added to the table. Through an iterative process while reviewing sources, we created, edited, and in some cases removed data base fields as variation in historical data types and their usefulness became better understood. The process resulted in our inclusion of 33 fields in the final dataset (Fig. 4). We held weekly meetings during this time, and regular discussions allowed us to refine database fields until they fit well with the types of data that existed in archives. Fields that were ultimately removed from the final database mainly involved fish-specific details such as sex, length, weight, and age since these types of metadata, while deemed important to capture at the outset of the project, were rarely found in primary sources. Any metadata we discovered related to fish-specific fields were entered into a general NOTES field (Fig. 4).

### Spatial accuracy

Every record in CORHIST includes coordinates (point locations) in decimal degrees (LAT_DD and LON_DD; WGS84; Fig. 4) that best represent a point for a historical observation or occurrence. In some cases, the coordinates represent a centroid of a larger area (i.e., a reef, shoal, shoreline, or embayment), and the additional metadata to describe the location is included in a location notes field (see LOC_NOTES, Fig. 4). Most point locations were determined using original location information found in primary sources, including dead reckonings and other compass bearings, direction and speed information from known landmarks and vessels, or degree, minutes, seconds, all of which can be converted to decimal degrees with varying spatial acuity. Dead reckonings, a historical navigation method that used direction (*e.g*., a 128-point compass rose), time, and approximated vessel speed (*e.g*., knots or later engine RPMs) to estimate distance travelled from known points, were the most frequently discovered location description in historical sources.

Point locations calculated using the dead reckoning method first converted the origin point (usually a port city) to a Universal Transverse Mercator (UTM) coordinate within the relevant UTM zone (e.g., 15–18 in the Basin) to standardize the eastings and northings in meters. To account for historical changes in magnetic declination over the expanded time frame of the source data, directions (compass bearing degrees) were adjusted by acquiring the magnetic declination during the historical date and approximate location of the source record. The magnetic declination was subtracted (west declinations) or added (east declination) to the compass bearing to standardize all source dead reckonings to the current map and used with the distance travelled (km) to calculate the final destination Easting and Northing coordinate in meters. All conversions from eastings/northings into latitude/longitude decimal degrees were made using the map datum WGS84 and Montana State University’s Geographic Units conversion tool (https://rcn.montana.edu/Resources/Converter.aspx).

When lower-resolution direction and distance information was discovered in an original source (*e.g*., “12 km NE of Harbor Beach Light, Lake Huron”), a 128-point compass rose table with degrees and associated decimals was referenced along with the Measure Distance tool in ArcGIS Pro (datum: WGS84) to determine a point in decimal degrees. Path Net Bearing and Path Net Distance were utilized to ensure the correct degrees for direction and correct distance measured, respectively. When degrees, decimal minutes, or when degrees, minutes, seconds, were provided in an original source, these were converted to decimal degrees (datum: WGS84) with Montana State University’s Geographic Units conversion tool (http://rcn.montana.edu/Resources/Converter.aspx).

Occasionally a historical map was discovered in an original source that described important habitats, spawning locations, or hotspots for fishing activity. Locations on these maps were converted to decimal degrees using Georeferencing tools in ArcMap 10.8 and ArcGIS Pro through a multi-step rubbersheeting approach as a secondary check to verify locations generated from the georeferencing approach mentioned above matched the original map. First, a scan of the map, usually a PDF converted to a TIFF, was added to a new GIS project. A shoreline shapefile was added to pair with the image file. As an example, for Lake Michigan, a shoreline shapefile would be accessed and added from the Michigan Center for Geographic Information (https://gis-michigan.opendata.arcgis.com/). Other shoreline vectors used in this study came from NOAA’s Historical Medium Resolution Shoreline (datum: NAD83; https://shoreline.noaa.gov/med-res.html). Both map layers would then be fit to display at the same time and control points were added. If a grid or any symbols with given coordinates were shown on the original map, those would be used to add control points. However, in most historical maps no grid or coordinates were given. In these cases, known landmarks such as islands, river mouths, and lighthouses were used to add control points to the scanned map. Approximately 6 control points were added to achieve the best alignment between the base TIFF layer and base shoreline layer, at which point the link table would be viewed to check the error and residuals for each control point. Residuals of 0.05 pixels or lower were considered acceptable. Once alignment was achieved, the newly transformed historical map was saved as a raster for further geocoding of points of interest. Each point of interest was then digitized manually by clicking on each map symbol using the Point option in the Create Features window of ArcMap. Since many of the symbols on historical maps are large and often hand-drawn, every attempt was made to click the center of each symbol to be as accurate as possible. Once all point digitization was complete, decimal degrees for each symbol were compared to map locations that were previously georeferenced using descriptions found in original text to confirm accuracy of original point locations.

For records where location information was lower quality but still acceptable (*e.g*., “out from Port Huron,” or “just west of Thunder Bay Lighthouse”) additional sources such as historical maps^40^, records of historical fishing ground locations, or other sources containing reef locations or substrate information (data layers available at https://www.glahf.org/, not of this dataset) were used to tag a point as accurately as possible. Publicly available bathymetry data were used in ArcGIS Pro to confirm depth data in relation to point data when original depth information was provided in primary sources. These layers were developed in a cooperative effort between the NOAA National Geophysical Data Center’s Marine Geology and Geophysics Division and the NOAA Great Lakes Environmental Research Laboratory; (https://services.arcgis.com/As5CFN3ThbQpy8Ph/arcgis/rest/services/Great_Lakes_Bathymetry/FeatureServer). Where historical town or port names were given in original sources that are no longer recognized names, historical maps were consulted to ensure the correct place was used for georeferencing, and the original and updated place name was added to the dataset. Historical Great Lakes maps were accessed and studied using online collections including the University of Michigan Library’s digital collections (available at https://www.lib.umich.edu/) and the University at Buffalo online research libraries (available at https://research.lib.buffalo.edu/greatlakes/historicalmaps), as well as the Biodiversity Heritage Library (https://www.biodiversitylibrary.org/). When location information was lacking sufficient detail for georeferencing (*e.g*., only a lake name given, or no originating port given), the data were excluded since no reliable coordinate could be assigned.

### Species

Matching currently accepted taxonomy with historical names for coregonines was especially challenging in this project, as every region, culture, and decade tended to carry with it a suite of new and different common names associated with these fishes. This coregonine-specific dataset includes 11 accepted species names, along with several names that have been described and published but do not have accompanying Integrated Taxonomic Information System (ITIS) numbers. These additional names were included in the SPECIES reference table for posterity. The absence of an ITIS number is indicated with a null value in the ITIS field of the SPECIES reference table included with this dataset.

Because high taxonomic accuracy is critical to this study, our first step was to create a list of alternate names we identified in historical literature, including a multitude of common and scientific names, as well as names in multiple languages. The alternate name and corresponding ITIS accepted CORHIST species code were added to the list, along with any notes related to the source, origin, or context of the alternative name, when an alternative name was identified in text and associated species confirmed in ITIS (Supplemental Table 1). Evidence for assigning an accepted scientific name to an alternate name typically came from a photograph or illustration, morphological description, or by expert evaluation from the CORHIST team. Common and scientific names for many coregonines regularly change across time and regions, making many of the alternate names on the list highly context dependent. While the alternate names list was useful when conducting archival research including querying online catalogues and reading physical sources, it is by no means a definitive list of names that correspond to one single species. Nearly 140 additional names were discovered and documented during this process (Supplemental Table 1).

Often an original source had ample evidence to assign an ITIS accepted species code to each alternate name, usually thanks to an accompanying illustration, morphometric description, or photo in the original source. In many cases the currently excepted ITIS name was used in the original source making the alternate names list obsolete. If it was impossible to attribute an ITIS accepted scientific name to an alternate name found in a record, yet the team was confident from evidence in the text that the species was a coregonine, a genus-level code was attributed to the record instead of a species-specific code (i.e., SPECIES code 999: *Coregonus* or *Prosopium* spp.).

## Data Records

The CORHIST database, including a main table, all reference tables, and all associated metadata are available in CSV and XML formats to the public on ScienceBase (10.5066/P13PPGAH)^41^. Every data record in CORHIST is linked to its original citation or source (see SOURCE reference table, Fig. 4). A relational database structure was created for CORHIST using Oracle (Oracle Database Solutions), and later refined to its current structure using Microsoft Access (Fig. 4). Refinements included removing reference tables that were not being used, as well as removing additional relational tables and fields that either added redundancy or were never populated with data during the review and indexing of historical sources. Following the May 9, 2013, Office of Management and Budget (OMB) memorandum “Open Data Policy—Managing Information as an Asset,” (https://www.whitehouse.gov/wp-content/uploads/legacy_drupal_files/omb/memoranda/2013/m-13-13.pdf) data published by U.S. Geological Survey (USGS) authors should be provided as open source (non-proprietary) format along with extensible metadata. As such the CORHIST database was published in CSV files and accompanied by an XML formatted metadata file. This is the first version of the CORHIST database, it has been peer-reviewed, and updated versions will be created if/when new sources or data become available.

## Technical Validation

The Atlas of the Spawning and Nursery Areas of Great Lakes Fishes^20^, which still remains a primary source for spawning information for a wide range of Great Lakes fishes, is included in the CORHIST dataset and linked to that citation. To ensure records were not duplicated, the team processed (georeferenced, performed quality control and quality assurance, and indexed data and metadata) all coregonine records in the original Goodyear *et al*.^20^ atlas volumes, 1–14, prior to including them in CORHIST. Citations from Goodyear *et al*.^20^ were examined, locations descriptions were georeferenced, and georeferenced points were regularly summarized using ArcMap 10.8 and ArcGIS Pro to ensure points did not overlap between Goodyear *et al*.^20^ and novel historical records. In checking original citations in Goodyear *et al*.^20^, we discovered additional records or evidence that were not yet documented, and in those cases both the Goodyear *et al*.^20^ records and novel records from primary sources were added to CORHIST. Points were mapped and checked using ArcMap 10.8 and ArcGIS Pro to ensure that no points occurred on land. When depth information was given in the original source, points were mapped and checked against bathymetry data (https://www.ngdc.noaa.gov/mgg/greatlakes/) to ensure the point was mapped at the correct depth contour(s). We regularly compared points from atlases to the points discovered in primary sources to ensure minimal to no duplication of records occurred. In most cases, spawning atlases had significantly broader spatial resolution than the coordinates that our team georeferenced from a primary source, resulting in very little duplication of records occurring when comparing mapped points between sources. Spawning atlases often extrapolated spawning areas to create a larger polygon, usually encompassing entire reefs, shorelines, or island perimeters based on multiple sources including personal communications that could not be further researched by our team. If a duplicate record was discovered between secondary atlas sources and a primary source, the secondary atlas duplicate record was removed.

Following the USGS Survey Manual 502.8 - Fundamental Science Practices: Review and Approval of Scientific Data for Release (https://www.usgs.gov/survey-manual/5028-fundamental-science-practices-review-and-approval-scientific-data-release), these data and metadata were both reviewed before publication in the USGS repository ScienceBase (https://www.sciencebase.gov/catalog/). The XML metadata created for the CORHIST data release use the schema Federal Geographic Data Committee (FGDC) Biological Data Profile of the Content Standard for Digital Geospatial Metadata (CSDGM) (version FGDC-STD-001.1-1999). FGDC CSDGM XML can be created, updated, and viewed in the metadata editor Metadata Wizard, developed by the Data Management Team at the USGS Fort Collins Science Center (https://github.com/DOI-USGS/fort-pymdwizard/releases).

The metadata review starts with a validation of the schema using the USGS developed Metadata Parser (https://www.usgs.gov/mp/). The Parser checks that the metadata elements are ordered properly and that required fields are filled in. A set of baseline checklists were created by the USGS Metadata Reviewers Community for metadata and data review (https://www.usgs.gov/media/files/metadata-review-checklist-0). Some of the main points reviewed in the XML metadata, which are reflected in the metadata checklist, include:

- Data quality and process steps are defined and transparent
- All tables and columns being published are defined and that relationships between tables are described
- Codes are defined
- Null and unknown values are described
- Units of measure are provided where applicable

**Fig. 5:**
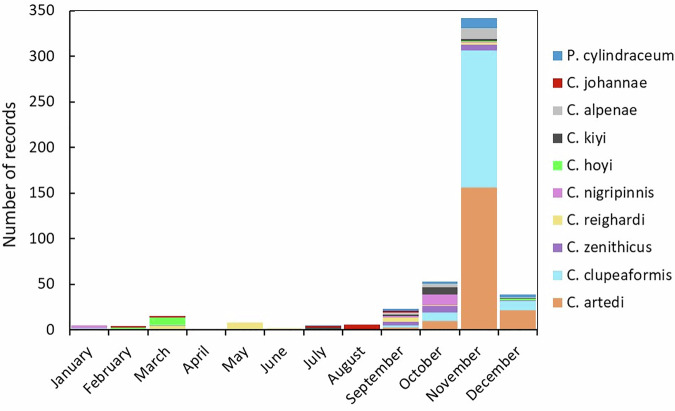
A stacked column graph showing the number of records designated as Spawning and Possible Spawning (combined) for each species and month in CORHIST.

We examined maximum and minimum values and unique sets of values (*e.g*., lists of higher-than-average values, or lower-than-average values) recorded in each column of a CSV. We checked that this information matched the XML metadata and that outliers or misspellings were addressed. We screened for data duplication using “Find Duplicates” queries. Where depth information was given in original records, associated occurrence points from those records were mapped and checked with a bathymetry layer (https://www.ncei.noaa.gov/products/great-lakes-bathymetry) to ensure the depth of the location matched with the original description. While we utilized spell check features in Microsoft Excel and Access, we also aimed to keep as true and accurate to the historical record as possible. In some cases we directly transcribed location spelling as seen in the original source into the LOC_NOTES text field to ensure that no historical vernacular was lost once a record was indexed. We graphed all spawning records by month and species to check that records were near or within the spawning month ranges for each species generally described in the literature (Fig. 5).

## Usage Notes

Users are advised to read the dataset’s metadata thoroughly to understand appropriate use and data limitations (available at 10.5066/P13PPGAH). Historical information related to spawning occurrence for fishes is diverse in detail, especially related to location, timing, and physiology (*e.g*., spawning status, life stage). Users of these data should understand that while most data for each record were transcribed exactly as seen in the original source, values in fields such as SPAWNING_CLASSIFICATION were assigned using evidence-based designations (assumptions based on data) by our research team. The goal of our team was to research, interpret, and extract spawning evidence from original records as systematically and accurately as possible. Like all historical species datasets, true species occurrence in most cases was likely much broader than what our data team was able to discover in documents. Cultural shifts in society, poor preservation or loss of documents, commercial and recreational preferences for certain species over others, and many other factors creates waves of biases through time and across regions for record availability. Major climatic shifts such as drought and flooding likely impacted spawning areas over time, and specific habitats or regions highlighted in CORHIST as once prominent spawning areas cannot be assumed unchanged in contemporary times. Exploitation and degradation of fish stocks in the Great Lakes occurred at different rates in each lake—Lake Ontario and Lake Erie were exploited much earlier than the upper lakes^24^. The availability of historical information is likely biased regionally, with fewer records on historic fisheries available for the lower lakes due to earlier impacts. While our team made every attempt to find and review available records, certain regions of the Great Lakes could not be visited in person, especially during the global pandemic, leaving many archives and original documents unsearched.

Our team would like users of these data to understand that CORHIST is currently deficient in its inclusion of IEK. Incorporating a combination of non-Indigenous and Indigenous sources and ways of knowing can yield more diverse and representative data for spawning locations, seasons, habitats, and behaviors^19,42^. A goal of our team is to continue to build long-standing relationships with Indigenous communities and embrace Two-Eyed Seeing—learning to see the strengths and value of IEK through one lens and mainstream knowledge (*i.e*., Western or positivist science) through the other as a guiding framework for knowledge generation toward everyone’s benefit^43^. Inclusion of IEK and co-production to enhance CORHIST could lead to strong partnerships and empower Indigenous resource managers to continue governing natural resources throughout Tribal and First Nations territories.

Finally, records in CORHIST are not meant to illustrate general population range or extent. As mentioned above, many offshore areas in the largest lakes were not sampled or fished, and still rarely are today. Large spatial gaps with no data in CORHIST do not necessarily mean the target species did not utilize those locations for spawning or nursery areas, and the potential importance of those locations remains uncertain. The CORHIST database is not complete, nor is it definitive, and every effort will be made to update the database upon discovery of new records and sources.

## Supplementary information

Suplemental Table 1

## Data Availability

The CORHIST database^41^, along with all reference tables and metadata, have been peer-reviewed following the U.S. Geological Survey’s Data Review Process (https://www.usgs.gov/sciencebase-instructions-and-documentation/data-release-process) and all data and metadata are published on ScienceBase: 10.5066/P13PPGAH. This work is marked with Creative Commons Zero v1.0 Universal (https://creativecommons.org/publicdomain/zero/1.0/).

## Code availability

The authors declare that no custom code was generated for this manuscript.

## References

[1] Jessen, T. D., Ban, N. C., Claxton, N. X. & Darimont, C. T. Contributions of Indigenous Knowledge to ecological and evolutionary understanding. *Frontiers in Ecology and the Environment* **20**, 93–101, 10.1002/fee.2435 (2022).

[2] Pooley, S. Descent with modification: Critical use of historical evidence for conservation. *Conservation Letters* **11**, e12437 (2018).

[3] Szabó, P. Why history matters in ecology: an interdisciplinary perspective. *Environmental Conservation* **37**, 380–387, 10.1017/S0376892910000718 (2010).

[4] Bronte, C. R. & Sitar, S. P. Harvest and Relative Abundance of Siscowet Lake Trout in Michigan Waters of Lake Superior, 1929‐1961. *Transactions of the American Fisheries Society* **137**, 916–926, 10.1577/t07-096.1 (2008).

[5] Ferrer-Paris, J. R., Sánchez-Mercado, A., Rodríguez-Clark, K. M., Rodríguez, J. P. & Rodríguez, G. A. Using limited data to detect changes in species distributions: insights from Amazon parrots in Venezuela. *Biological Conservation* **173**, 133–143 (2014).

[6] Fortibuoni, T. *et al*. Fish and fishery historical data since the 19th century in the Adriatic Sea, Mediterranean. *Scientific Data* **4**, 170104, 10.1038/sdata.2017.104 (2017). 28895949 10.1038/sdata.2017.104 PMC5595044

[7] Kao, Y.-C., Bunnell, D. B., Eshenroder, R. L. & Murray, D. N. Describing historical habitat use of a native fish—Cisco (*Coregonus artedi*)—in Lake Michigan between 1930 and 1932. *PLOS One* **15**, e0231420 (2020). 32267898 10.1371/journal.pone.0231420 PMC7141674

[8] Selgeby, J. H. Decline of Lake Herring (*Coregonus artedii*) In Lake Superior: An Analysis of the Wisconsin Herring Fishery, 1936–78. *Canadian Journal of Fisheries and Aquatic Sciences* **39**, 554–563, 10.1139/f82-079 (1982).

[9] Pauly, D. Anecdotes and the shifting baseline syndrome of fisheries. *Trends in Ecology and Evolution* **10**, 430 (1995). 21237093 10.1016/s0169-5347(00)89171-5

[10] Gadgil, M., Berkes, F. & Folke, C. in *Foundations of Socio-Environmental Research: Legacy Readings with Commentaries Part IV - Socio-Environmental Research in Ecology* (eds William R. Burnside *et al*.) 506–511 (Cambridge University Press, 1993).

[11] Jardine, T. D. Indigenous knowledge as a remedy for shifting baseline syndrome. *Frontiers in Ecology and the Environment* **17**, 13–14 (2019).

[12] McClenachan, L., Ferretti, F. & Baum, J. K. From archives to conservation: why historical data are needed to set baselines for marine animals and ecosystems. *Conservation Letters* **5**, 349–359, 10.1111/j.1755-263X.2012.00253.x (2012).

[13] Pauly, D. *Vanishing fish: shifting baselines and the future of global fisheries*. (Greystone Books Ltd, 2019).

[14] Alexander, K. E. *et al*. Gulf of Maine cod in 1861: historical analysis of fishery logbooks, with ecosystem implications. *Fish and Fisheries* **10**, 428–449, 10.1111/j.1467-2979.2009.00334.x (2009).

[15] Eshenroder, R. L. *et al*. Ciscoes (Coregonus, subgenus Leucichthys) of the Laurentian Great Lakes and Lake Nipigon. (Great Lakes Fishery Commission, 2016).

[16] Koelz, W. *Coregonid fishes of the Great Lakes* (U.S. Government Printing Office Washington, D. C., 1929).

[17] Page, L. M. *et al*. *Common and scientific names of fishes from the United States, Canada and Mexico*. Eighth Edition edn, (American Fisheries Society, 2023).

[18] Blackbird, A. J. *History of the Ottawa and Chippewa Indians of Michigan: A grammar of their language, and personal and family history of the author*. (The Ypsilantian Job Printing House, 1887).

[19] Duncan, A. T., Lauzon, R. & Harpur, C. An investigation into Saugeen Ojibway Nation-based ecological knowledge on the ciscoes (Coregonus spp.) of Lake Huron. *Journal of Great Lakes Research* **49**, S138–S147, 10.1016/j.jglr.2023.02.004 (2023).

[20] Goodyear, C. S., Edsall, T. A., Ormsby Dempsey, D. M., Moss, G. D. & Polanski, P. E. (ed U.S. Fish and Wildlife Service) (Washington, DC, 1982).

[21] Goodier, J. L. The nineteenth-century fisheries of the Hudson’s Bay Company trading posts on Lake Superior: A biogeographical study. *Canadian Geographies/Géographies canadiennes* **28**, 341–357, 10.1111/j.1541-0064.1984.tb01608.x (1984).

[22] Ransom, A. L. *et al*. Recolonization of lake whitefish river spawning ecotypes and estimates of riverine larval production in Green Bay, Lake Michigan. *Journal of Great Lakes Research* **47**, 213–225, 10.1016/j.jglr.2020.11.011 (2021).

[23] Dryer, W. R. & Beil, J. Life history of lake herring in Lake Superior. *Fish. Bull* **63**, 493–530 (1964).

[24] Bogue, M. B. *Fishing the Great Lakes: An environmental history, 1783–1933*. (University of Wisconsin Press, 2001).

[25] Brant, C. O. *Great Lakes sea lamprey: the 70 year war on a biological invader*. (University of Michigan Press, 2019).

[26] Christie, W. J. Changes in the Fish Species Composition of the Great Lakes. *Journal of the Fisheries Research Board of Canada* **31**, 827–854, 10.1139/f74-104 (1974).

[27] Lower, E. *et al*. The Great Lakes’ most unwanted: Characterizing the impacts of the top ten Great Lakes aquatic invasive species. *Journal of Great Lakes Research* **50**, 102365, 10.1016/j.jglr.2024.102365 (2024).

[28] Nalepa, T. F., Fanslow, D. L. & Lang, G. A. Transformation of the offshore benthic community in Lake Michigan: recent shift from the native amphipod Diporeia spp. to the invasive mussel *Dreissena rostriformis bugensis*. *Freshwater Biology* **54**, 466–479, 10.1111/j.1365-2427.2008.02123.x (2009).

[29] Koelz, W. *Fishing industry of the Great Lakes*. (US Government Printing Office, Washington, D.C., 1926).

[30] Smith, S. H. Factors of ecologic succession in oligotrophic fish communities of the Laurentian Great Lakes. *Journal of the Fisheries Board of Canada* **29**, 717–730 (1972).

[31] Bunnell, D. B. *et al*. A science and management partnership to restore coregonine diversity to the Laurentian Great Lakes. *Environmental Reviews* **31**, 716–738, 10.1139/er-2022-0109 (2023).

[32] Milner, J. W. Report on the fisheries of the Great Lakes: the result of inquiries prosecuted in 1871 and 1872. *Report of the US Commissioner on Fish and Fisheries for*, 1–78 (1872).

[33] Loftus, D. H. *Interviews with Lake Huron commercial fishermen*. 1–80 (Lake Huron Fisheries Assessment Unit, Ontario Ministry of Natural Resources, Owen Sound, ON, 1980).

[34] Pennino, M. G. *et al*. Fishery-dependent and -independent data lead to consistent estimations of essential habitats. *ICES Journal of Marine Science* **73**, 2302–2310, 10.1093/icesjms/fsw062 (2016).

[35] Brown‐Peterson, N. J., Wyanski, D. M., Saborido‐Rey, F., Macewicz, B. J. & Lowerre‐Barbieri, S. K. A Standardized Terminology for Describing Reproductive Development in Fishes. *Marine and Coastal Fisheries* **3**, 52–70, 10.1080/19425120.2011.555724 (2011).

[36] Brant, C. O. *et al*. *Gap Analysis: A proposed methodology to describe and map historical and contemporary populations and habitats*. 30. https://www.greatlakesciscoes.org/restoration-framework/planning/ (2023).

[37] Ackiss, A. S. *et al*. *Delineating spatial units for coregonine conservation, restoration, and stewardship*. 30. https://www.greatlakesciscoes.org/restoration-framework/planning/ (2023).

[38] Coberly, C. E. & Horrall, R. M. *Fish spawning grounds in Wisconsin waters of the Great Lakes*. 1–43 (University of Wisconsin Sea Grant Institute, 1980).

[39] Organ, W., Towns, G., Walter, M., Pelletier, R. & Riege, D. Past and presently known spawning grounds of fishes in the Michigan coastal waters of the Great Lakes. *Michigan Department of Natural Resources*. **577** (Aquatic Systems, Inc., Ludington, MI, 1979).

[40] Woodford, A. M. *Charting the inland seas: a history of the US Lake Survey*. (Wayne State University Press, 1994).

[41] Brant, C. O. *et al*. *ScienceBase, U.S. Geological Survey*10.5066/P13PPGAH (2024).

[42] Moller, H., Berkes, F., Lyver, P. O. B. & Kislalioglu, M. Combining Science and Traditional Ecological Knowledge: Monitoring Populations for Co-Management. *Ecology and Society* **9** (2004).

[43] Reid, A. J. *et al*. “Two-Eyed Seeing”: An Indigenous framework to transform fisheries research and management. *Fish and Fisheries* **22**, 243–261, 10.1111/faf.12516 (2021).

