# Supplementary material for: Two hundred years of historical spawning and nursery data for coregonine fishes in the Laurentian Great Lakes
Source: Sci Data. 2026 May 7;13:711. doi: 10.1038/s41597-026-06974-1 (PMC13153148; doi:10.1038/s41597-026-06974-1)
Supplement: Supplementary file 1 — Suplemental Table 1 [file 41597_2026_6974_MOESM1_ESM.pdf]

SPECIES	Alternative name to query	Notes on source or origin
1	Argyrosomus artedi	Lesueur, 1818; Evermann and Smith, 1896
1	Argyrosomus cisco	Jordan, 1875
1	Argyrosomus eriensis	Jordan and Evermann, 1909
1	Argyrosomus huronius	Jordan and Evermann, 1909
1	Argyrosomus tullibee	Richardson, 1836; Evermann and Smith, 1896
1	Argyrosomus tullibee bisselli	Evermann and Smith, 1896
1	Coregonus tullibee bisselli	Bollman, 1889
1	Leucichthys artedi	Lesueur, 1818; Jordan and Evermann, 1911; Dymond, 1926
1	Leucichthys churchillensis	Fowler, 1948
1	Leucichthys cisco	Jordan and Evermann, 1911
1	Leucichthys cisco huronius	Jordan and Evermann, 1911
1	Leucichthys eriensis	Jordan and Evermann, 1909; Clemens, 1922
1	Leucichthys harengus	Richardson, 1836; Jordan and Evermann, 1911; Bays of Lakes Huron and Michigan
1	Leucichthys harengus arcturus	Jordan and Evermann, 1911
1	Leucichthys macropterus	Bean, 1916
1	Leucichthys manitoulinus	Jordan and Evermann, 1909; North Channel of Lake Huron
1	Leucichthys nueltinensis	Fowler, 1948
1	Leucichthys ontariensis	Jordan and Evermann, 1909; Jordan and Evermann 1911; Lake Ontario
1	Leucichthys supernas	Jordan and Evermann, 1909; Jordan and Evermann 1911; Lake Superior
1	Leucichthys tullibee	Richardson, 1836; Jordan and Evermann, 1911
1	Leucicthys artedi bisselli	Jordan and Evermann, 1911
1	Salmo (Coregonus) harengus	Richardson, 1836; Georgian Bay Lake Huron
1	Salmo artedi	Lesueur, 1818
1	Salmo harengus	Richardson, 1836
1	Salmo tullibee	Richardson, 1836
1	Lake Cisco	various Great Lakes ports
1	Lake Herring	various Great Lakes ports
1	Sisco	various Lake Erie ports
1	Michigan Herring	various Great Lakes ports
1	Michigan Blueback	various Great Lakes ports
1	Herring	various Great Lakes ports
1	Shore Herring	Lake Superior
1	Blueback	various Great Lakes ports
1	Greenback	various Great Lakes ports
1	Odoonibiins	Ojibwe Anishinaabemowin
1	Otoonapii	Ojibwe Anishinaabemowin
1	Kaviselik	Inuktitut
1	Mongrel Whitefish	Canadian Northwest
1	Tullibee	various Great Lakes ports
1	Cisco de lac	French Canadian
1	Corégone cisco	French Canadian

1	Coregono de artedi	Spain
1	Reef Herring	Lake Superior; Coberly and Horrall, 1980
1	Deep Herring	Lake Superior; Coberly and Horrall, 1980
1	Bottom Herring	Lake Superior; Coberly and Horrall, 1980
1	Mud Herring	Lake Superior; Coberly and Horrall, 1980
2	Salmo clupeaformis	Mitchill, 1818
2	Coregonus albus	Lesueur, 1818
2	Salmo labradoricus	Richardson, 1836
2	Coregonus labradoricus	Jordan and Evermann, 1902; Canada's Northwest Territory
2	Coregonus sapidissimus	Agassiz, 1850
2	Coregonus latior	Agassiz, 1850
2	Coregonus atikameg	Bajkov, 1933
2	Musquaw River whitefish	Goode, 1887
2	Whiting	northern New England, c. 1890s
2	Humpback Whitefish	various ports around Lake Erie
2	Bowback Whitefish	various ports around Lake Erie
2	Highback Whitefish	various ports around Lake Erie
2	Otsego Bass	Lake Otsego, NY
2	Gizzard Fish	Canada
2	Eastern Whitefish	Canada
2	Sault Whitefish	Canada
2	Grand Corégone	French Canadian
2	Corégone de lac	French Canadian
2	Inland Whitefish	Canada
2	Anâdlerk	Inuktitut
2	Qalupiaq	Inuktitut
2	Adikameg	Ojibwe Anishinaabemowin
2	Tittamingue	Métis
3	Argyrosomus zenithicus	Jordan and Evermann, 1909
3	Leucichthys zenithicus	Jordan and Evermann, 1909
3	Leucichthys entomophagus	Harper and Nichols, 1919
3	Lake Superior longjaw	Van Oosten, 1937
3	Light-back tullibee	various Great Lakes ports
3	Longjaw cisco	various Great Lakes ports
3	Pale-back tullibee	various Great Lakes ports
3	Short-jawed chub	Koelz, 1929; Hile and Deason, 1944
3	Cisco à mâchoires égales	French Canadian
4	Leucichthys reighardi	Koelz, 1924
4	Coregonus reighardi reighardi	Koelz, 1924
4	Greaser	Canada, Lake Ontario and perhaps other lakes
4	Reighard Cisco	various Great Lakes ports
4	Reighard Chub	various Great Lakes ports
4	Shortnose Chub	various Great Lakes ports
4	Cisco à museau court	French Canadian
4	Subchub	Koelz, 1920
5	Argyrosomus nigripinnis	Milner, 1874

5	Coregonus nigripinnis nigripinnis	Milner, 1874
5	Leucichthys nigripinnis regalis	Koelz, 1929
5	Coregonus nigripinnis regalis	Koelz, 1929
5	Black-fin	Koelz, 1929
5	Blackfin Whitefish	Koelz, 1929
5	Bluefin	Koelz, 1929
5	Blue-fin	Koelz, 1929
6	Argyrosomus hoyi	Milner, 1874
6	Coregonus osmeriformis	Bean, 1883; Seneca Lake, NY
7	Moon-eye	Koelz, 1921
7	Mooneye	Koelz, 1921
7	Waterbelly	Koelz, 1921
7	Leucichthys kiyi	Koelz, 1921
7	Leucichthys kiyi orientalis	Koelz, 1929, Lake Ontario
7	Coregonus kiyi orientalis	Koelz, 1929, Lake Ontario
7	Coregonus kiyi kiyi	upper Great Lakes
8	Leucichthys alpenae	Koelz, 1924; Lakes Michigan and Huron
8	Argyrosomus prognathus	Evermann and Smith, 1896; Lakes Michigan and Huron
8	Leucichthys johannae	Jordan and Evermann, 1911; Lakes Michigan and Huron
8	Longjaw	Koelz, 1929
8	Longjaw Chub	various Great Lakes ports
8	Cisco à grande bouche	French Canadian
9	Argyrosomus johannae	Wagner, 1910; Lake Michigan
9	Leucichthys johannae	Wagner, 1910; Koelz, 1929
9	Argyrosomus hoyi	Milner, 1874; Evermann and Smith, 1896; Lake Michigan
9	The Chub	Koelz, 1929; Lakes Michigan and Huron
10	Salmo cylindraceus	Pennant, 1784
10	Coregonus cylindraceus	Pennant, 1784
10	Salmo microstomus	Pallas, 1814
10	Coregonus quadrilateralis	Richardson, 1823
10	Prosopium quadrilaterale	Richardson, 1823
10	Salmo quadrilateralis	Richardson, 1823
10	Coregonus mongolicus	Warpachowski, 1900
10	Prosopium preblei	Harper and Nichols, 1919
10	Pilot	Koelz, 1920; 1929
10	Pilot Fish	various Great Lakes ports
10	Frost Fish	Adirondacks
10	Menominee	various Great Lakes ports
10	Menominee whitefish	various Great Lakes ports
10	Round Fish	various Great Lakes ports
10	Ménomini rond	French Canadian
10	Okeugnak	Inuktitut
10	Savigunac	North America

10	Shad Waiter	Noth America, east coast
10	Grayback	various Great Lakes ports
10	Blackback	Lake Michigan
10	Chivey	Maine
10	Chateaugay shad	Chateaugay Lake
11	Coregonus coulterii	Eigenmann and Eigenmann, 1892
11	Prosopium snyderi	Myers, 1932
11	Brownback Whitefish	Canada
11	Coulter's Whitefish	North America
11	Ménomini Pygmée	French Canadian
